# Value of nerve root sedimentation sign in diagnosis and surgical indication of lumbar spinal stenosis

**DOI:** 10.1186/s12891-023-06459-x

**Published:** 2023-04-28

**Authors:** Guizhen Qian, Yanshuang Wang, Jiarong Huang, Dehua Wang, Chongchang Miao

**Affiliations:** Department of Radiology, The Affiliated Lianyungang Hospital of Xuzhou Medical University, 6# Zhenhua East Road, Haizhou District, Lianyungang, 222002 Jiangsu China

**Keywords:** Nerve root sedimentation sign, Lumbar spinal stenosis, Magnetic resonance imaging, Diagnosis

## Abstract

**Background:**

Lumbar spinal stenosis (LSS) is a prevalent and disabling cause of low back and leg pain in elderly people and nerve root sedimentation sign (NRSS) has been demonstrated to have high sensitivity and specificity in diagnosing LSS in selected patients. The purpose of this study was to investigate the diagnosis of LSS and the predictive value of NRSS.

**Methods:**

The clinical and imaging data of 176 patients diagnosed with LSS and 156 patients with non-specific low back pain (LBP) were analyzed retrospectively. Transverse magnetic resonance images (MRI) of the narrowest spinal canal in all patients were acquired and graded by two experienced doctors using the Braz classification, Schizas classification and Chen Jia classification. Receiver operating curve (ROC) was used to compare the diagnostic efficacy of the three classifications. Univariate and multivariate logistic regression models were established to predict the surgical indications of LSS patients.

**Result:**

The diagnostic efficacy of Schizas classification (AUC:0.943; 95%CI:0.918,0.969) and Chen Jia classification (AUC:0.942; 95%CI:0.918,0.966) was significantly higher than that of Braz classification (AUC:0.853; 95%CI:0.808,0.898). Chen Jia classification had the highest correlation with the degree of dural sac cross-sectional area (DCSA) stenosis. In the multivariate analysis of LSS surgical indications, Chen Jia classification (odds ratio [OR], 2.127; 95%CI:1.596,2.835), DCSA (OR,0.398; 95%CI:0.169,0.802) and intermittent claudication (OR,9.481; 95%CI:3.439,26.142) were associated with surgical indications.

**Conclusion:**

Among the three types, it is found that Chen Jia classification has better diagnostic efficacy in differentiating LSS from LBP. In addition, Chen Jia classification is simple to be implemented in clinical practice and has high clinical application value. Hence, Chen Jia classification can be used as an effective surgical treatment indicator for LSS patients.

## Background

Lumbar spinal stenosis (LSS) is a degenerative disease of the lumbar spine that occult commonly in the elderly. It is due mainly to the reduction of the anatomical space of the nerves and blood vessels in the lumbar spinal canal and clinically, it often presents as persistent low back pain, sacral pain or neurogenic intermittent claudication and so on [1]. One study estimated worldwide there are approximately 103 million individuals suffering from LSS [2] and is one of the most common reasons for performing spinal surgery in patients over 65 years of age [3]. Currently, the diagnosis of LSS depends mainly on clinical symptoms and imaging data [4]. The most objective method to evaluate anatomic spinal stenosis through imaging data is by using the dural sac cross-sectional area (DSCA) evaluation [5]. However, DCSA measurement is not easy to implement in clinical practice. Therefore, a well-defined and simple morphological classification for assessing the severity of anatomical spinal stenosis is very important. In 2010, Braz et al. [6] first proposed nerve root sedimentation sign (NRSS) as a method for assisting clinical diagnosis of LSS [7–9]. There are three types of magnetic resonance imaging (MRI) based nerve root classifications, namely Braz classification, Schizas classification [10] and Chen Jia classification [11]. Braz classification is simple but can easily cause false positive results [12, 13], while the other two methods are slightly more complex and are difficult to implement in clinical practice. Hence, the main purpose of this study was to explore the value of NRSS based on the three classifications method for differentiating the diagnosis of LSS and non-specific low back pain (LBP) and predicted the surgical indications for patients with LSS.

## Methods

This is a retrospective study. The study protocol was approved by ethics committee and the requirement for informed consent was waived.

### Patients

The study data used for the retrospective analysis of patients with low back pain or intermittent claudication was collected from January to December 2021. The inclusion criteria are (1) age > 45 years old; (2) MRI examination of lumbar spine due to low back pain or intermittent claudication; (3) the clinical diagnosis is LSS or LBP. The exclusion criteria: (1) history of lumbar surgery; (2) complication with spinal tumor, spinal trauma and spinal infection; (3) poor image quality or incomplete information. All clinical and imaging data of patients were collected at the time of diagnosis. The symptoms of patients with lumbar spinal stenosis did not improve or even worsen within 3–6 months or patients who developed loss of bowel or bladder function or has rapidly progressiveleg weakness will be treated by surgery.

### MRI protocol

Philips Ingenial 3.0 T second generation MR scanner was used for acquiring T2WI transverse plane MRI of the spine. TSE sequence with TR 2000- 5000 ms, TE 120 ms, echo chain length 25, bandwidth 185.1 Hz, field of view (FOV) 160 × 179 mm, slice thickness 4 mm, gap spacing 0.4 mm, matrix 268 × 216, number of excitation (NEX) 1.6 were used and the transverse plane selection was positioned in the median sagittal position, where the central line was positioned parallel to the bisector of intervertebral space or intervertebral angle, and 3–5 slices were continuously scanned from the head to foot direction.

### Image analysis

**Fig. 1:**
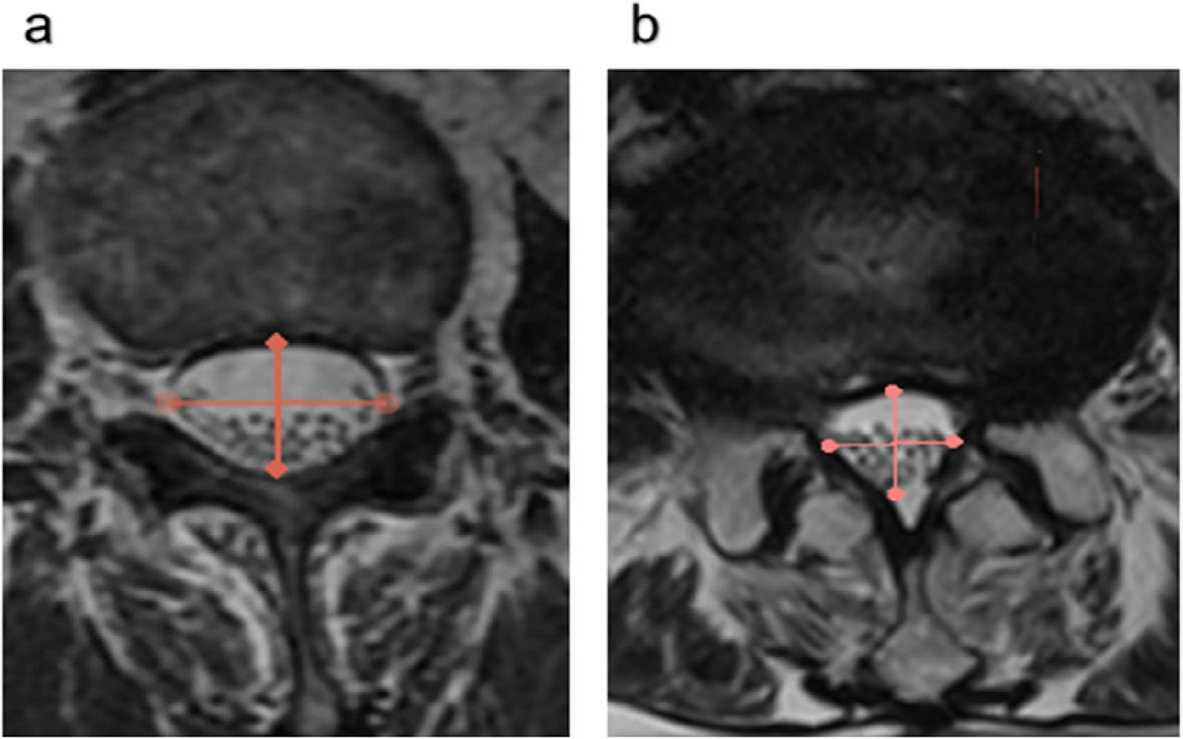
Braz classification (using vertical bisector of median sagittal diameter of dural sac cross section): (**a**) negative: the nerve root settles on the dorsal side of the dural sac under the action of gravity; (**b**)positive: the nerve roots are suspended, dispersed in the dural sac, or even floating to the ventral side of the dural sac

Braz classification: With reference to the T2WI transverse plane MRI, under the influence of the gravitational force, the negative nerve root sedimentation sign is identified as the nerve root settles on the dorsal side of the dural sac under the action of gravity (except the two nerve roots leaving the dural sac); On the contrary, except for the two nerve roots leaving the dural sac, if the other nerve roots are suspended, dispersed in the dural sac, or even floating to the ventral side of the dural sac, they are identified as positive nerve root sedimentation sign (Fig. 1).

**Fig. 2:**
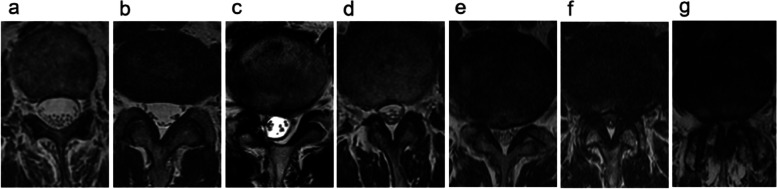
Schizas classification: (**a**) Grade A1: the rootlets lie dorsally and occupy less than half of the dural sac area; (**b**) Grade A2: the rootlets lie dorsally, in contact with the dura but in a horseshoe configuration; (**c**) Grade A3: the rootlets lie dorsally and occupy more than half of the dural sac area; (**d**) Grade A4: the rootlets lie centrally and occupy the majority of thedural sac area; (**e**) Grade B: the rootlets occupy the whole of the dural sac, but they can still be individualized; (**f**) Grade C: no rootlets can be recognized, here is epidural fat present posteriorly; (**g**) Grade D: in addition to no rootlets being recognizable there is no epidural fat posteriorly

Schizas classification: With reference to the T2WI transverse plane MRI, Schizas classification is based on the ratio of cerebrospinal fluid (CSF) to cauda equina nerve bundle in the image. Grade A1: the rootlets lie dorsally and occupy less than half of the dural sac area. Grade A2: the rootlets lie dorsally, in contact with the dura but in a horseshoe configuration. Grade A3: the rootlets lie dorsally and occupy more than half of the dural sac area. Grade A4: the rootlets lie centrally and occupy the majority of thedural sac area. Grade B: the rootlets occupy the whole of the dural sac, but they can still be individualized and some CSF is still presented giving a grainy appearance to the sac. Grade C: no rootlets can be recognized, the dural sac is shown as homogeneous gray signal with no CSF signal visible, and here is epidural fat present posteriorly. Grade D: in addition to no rootlets being recognizable there is no epidural fat posteriorly (Fig. 2).

**Fig. 3:**
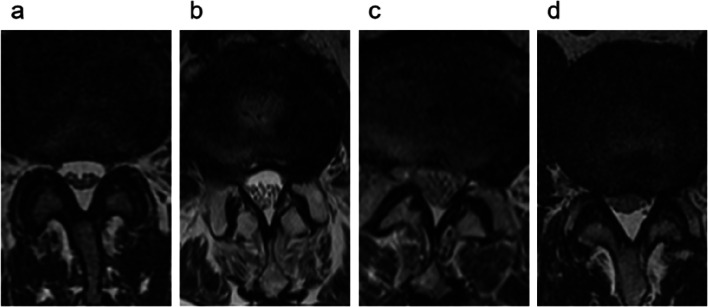
Chen Jia classification (**a**) negative:cauda equina nerve bundle is mainly located on the dorsal side of dural sac and the area of nerve bundle is less than 1/2 of dural sac; (**b**) Grade a: the area of cauda equina nerve bundle is greater than 1/2 of dural sac area; (**c**) Grade b: cauda equina nerve bundle occupies all the area of dural sac, but the fascicular structure of nerve can still be seen (**d**) Grade c: uniform gray signal in dural sac, unable to distinguish cerebrospinal fluid and nerve bundle

Chen Jia classification: With reference to the T2WI transverse plane MRI, negative classfication: cauda equina nerve bundle is mainly located on the dorsal side of dural sac, some nerve bundles can be distributed on the ventral side and the area of nerve bundle is less than 1/2 of dural sac; Grade a: the area of cauda equina nerve bundle is greater than 1/2 of dural sac area; Grade b: cauda equina nerve bundle occupies all the area of dural sac but the fascicular structure of the nerve can still be seen; Grade c: uniform gray signal in dural sac with undistinguishable CSF and nerve bundle (Fig. 3).

For measuring the area of the dural sac at the narrowest level on the MRI, 3D slicer 4.11 software is used. The lumbar spinal canal is defined according to the original proposed NRSS study, DCSA > 120 mm^2^ and DCSA < 80 mm^2^ criteria, for determining nonspecific LBP and LSS. The degree of stenosis is defined as no stenosis (> 120 mm^2^), mild stenosis (100–120 mm^2^), moderate stenosis (80–100 mm^2^) and severe stenosis (< 80 mm^2^) respectively [6, 14].

### Statistical analysis

For this study, IBM SPSS 26.0 software is used for all statistical analysis, and *p* < 0.05 is regarded as statistically significant. The data conforming to the normal distribution are expressed as the mean ± standard deviation. Two independent samples t-test are used for comparison between two groups, and one-way ANOVA variance analysis is used for comparison between multiple groups. Data that do not conform to the normal distribution are expressed as the median ± interquartile range and Wilcoxon test is used for comparisons between two groups. Count data are compared using the chi-square test or Fisher exact test and Kappa consistency test is used to evaluate the consistency of the independent grading results of the two physicians.The receiver operating characteristic curve (ROC) is used to evaluate the efficacy. The correlation between the three classifications and the degree of stenosis of DCSA is analyzed by Kendall's tau-b. In order to evaluate the surgical indications of LSS patients, a multivariate Logistic model is established, which included all clinical and imaging variables related to the surgical indications of LSS patients.

## Results

### Clinical and imaging data of patients

According to the above criteria, 332 patients are recruited in this study, which include 133 males (40.1%) and 199 females (59.9%), whose age ranged from 45 to 88 years with an average of (62.1 ± 9.5) years. Among the 332 patients, 156 (47.0%) are LBP patients, 176 (53.0%) are LSS patients, and 83 (47.2%) are LSS patients who has surgical intervention performed Table 1 summarizes the clinical data between patients with LBP and LSS. The age, VAS and pain time of LSS patients are significantly higher than those of LBP patients (mean age 65.8 years ± 8.7 vs 57.8 years ± 8.6, *p* < 0.01, the mean VAS is 2.02 ± 0.85 vs 1.36 ± 0.54, *p* < 0.01 and the mean pain time is 201.1 days ± 301.7 vs 57.9 days ± 134.3, *p* < 0.01). Among the LSS patients, male (59.7%) patients are significantly more likely to choose surgical treatment than female patients (38.5%) (*p* = 0.005), and the incidence of intermittent claudication and lower extremity pain in LSS surgical treatment group is significantly higher than that in LSS conservative treatment group patients (*p* < 0.01). As shown in the imaging data of Table 2, the three classifications and DCSA are significantly different between the LBP and LSS groups, as well as between the LSS conservative treatment group and the surgical treatment group (*p* < 0.001).

**Table 1:** Clinical characteristics of study samples

				LSS		
	LBP (*n* = 156)	Total LSS (*n* = 176)	*p*	LSS with conservative treatment (*n* = 93)	LSS with surgical treatment (*n* = 83)	*p*
age/y	57 (51,63)	66 (59,73)	< 0.001*	66.43 ± 9.36	65.18 ± 7.97	0.389^▲^
Gender			0.737			0.005
Female	95 (60.9)	104 (59.1)		64 (68.8)	40 (48.2)	
male	61 (39.1)	72 (40.9)		29 (31.2)	43 (51.8)	
VAS	1 (1,2)	2 (2,2)	< 0.001*	2 (2,2)	2 (2,2)	0.013*
Pain time			< 0.001			0.926
< 6w	72 (46.2)	32 (18.2)		16 (17.2)	15 (18.1)	
6-12w	15 (9.6)	14 (8.0)		7 (7.5)	7 (8.4)	
> 12w	69 (44.2)	130 (73.9)		70 (75.3)	61 (73.5)	
Back pian	153 (98.1)	159 (90.3)	0.003	87 (93.6)	72 (86.8)	0.127
Sacral pain	6 (3.9)	4 (2.3)	0.397	3 (3.2)	1 (1.2)	0.703
Hip pain	2 (1.3)	24 (13.6)	< 0.001	16 (17.2)	8 (9.6)	0.144
Lower limb pain	45 (28.9)	154 (87.5)	< 0.001	75 (80.7)	79 (95.2)	0.004
Intermittent rupture	1 (0.6)	133 (75.6)	< 0.001	55 (69.9)	78 (94.0)	< 0.001
Lower linb numbness	11 (7.1)	95 (54.0)	< 0.001	45 (48.4)	50 (60.2)	0.115

Except where indicated, the statistical method is chi-square test or Fisher exact test. *Wilcoxon test; ▲ t-test of two independent samples

*VAS* visual analogu scale

**Table 2:** Univariate analysis of image data of study samples

				LSS		
	LBP (*n* = 156)	Total LSS (*n* = 176)	*p*	LSS with conservation treatmengt (*n* = 93)	LSS with surgical treatment (*n* = 83)	*p*
DCSA	157.40 (127.31,187.50)	72.32 (127.31,187.50)	< 0.001*	82.95 (60.97,104.86)	53.20 (31.60,89.06)	< 0.001*
Braz classification			< 0.001			0.002
negative	119 (76.3)	10 (5.7)		10 (10.8)	0	
positive	37 (23.7)	166 (94.3)		83 (89.2)	83 (100)	
Chen Jia classification			< 0.001			< 0.001
negative	137 (87.8)	14 (8.0)		13 (14.0)	1 (1.2)	
a	17 (10.9)	30 (17.0)		25 (26.9)	5 (6.0)	
b	2 (1.3)	54 (30.7)		32 (34.4)	22 (26.5)	
c	0	78 (44.3)		23 (24.7)	55 (66.3)	
Schizas classification			< 0.001			< 0.001
A1	68 (43.6)	7 (4.0)		7 (7.5)	0	
A2	70 (44.9)	7 (4.0)		6 (6.5)	1 (1.2)	
A3	15 (9.6)	27 (15.3)		22 (23.7)	5 (6.0)	
A4	2 (1.3)	14 (8.0)		6 (6.5)	8 (9.6)	
B	1 (0.6)	42 (23.9)		28 (30.1)	14 (16.9)	
C	0	58 (33.0)		23 (24.7)	35 (42.2)	
D	0	21 (11.9)		1 (1.1)	20 (24.1)	

Except where indicated, the statistical method is chi-square test or Fisher exact test. *Wilicoxon test; *DCSA* dural sac cross-sectional area

### Consistency test of the three classifications

In this study, two experienced doctors are recruited for determining the three classifications of all patients, and after an interval of four weeks was again asked to re-perform the determination. It is found that the inter-observation and intra-observer Kappa values are greater than 0.75 (*p* < 0.001) (Table 3), suggesting that the classification is highly reliable and repeatable.

**Table 3:** Three classifications consistency test

	Braz classification	Chen Jia classification	Schizas classification
K1	0.752	0.856	0.846
K2	0.811	0.860	0.850

*K1* intraobserve, *K2* interobserve

### The advantage of using three classifications for diagnosing LSS and LBP

The AUC of Chen Jia classification is 0.942 (95% CI:0.918, 0.966) and the AUC of Schizas classification is 0.943 (95% CI:0.918, 0.969), which is significantly higher than Braz classification of 0.835 (95% CI:0.808, 0.898), with a statistically significant difference of *p* < 0.05 (Table 4). The diagnostic efficacy of Chen Jia classification and Schizas classification for LSS and LBP is significantly higher than that of Braz classification. There is no significant difference in the diagnostic efficacy between Chen Jia and Schizas classifications (*p* > 0.05), so the diagnostic accuracy of the two classifications for LSS and LBP is consistent.

**Table 4:** The diagnostic efficacy of three classifications

	AUC (95%CI)	Cut-off value	Sensitivity	Specificity
Braz classification*	0.853 (0.808,0.898)	——	94.3%	76.3%
Chen Jia classification*	0.942 (0.918,0.966)	> GradeA	92.0%	88.5%
Schizas classification*	0.943 (0.918,0.969)	> GradeA3	92.0%	87.8%

^*^p < 0.05

*AUC* area under curve

**Fig. 4:**
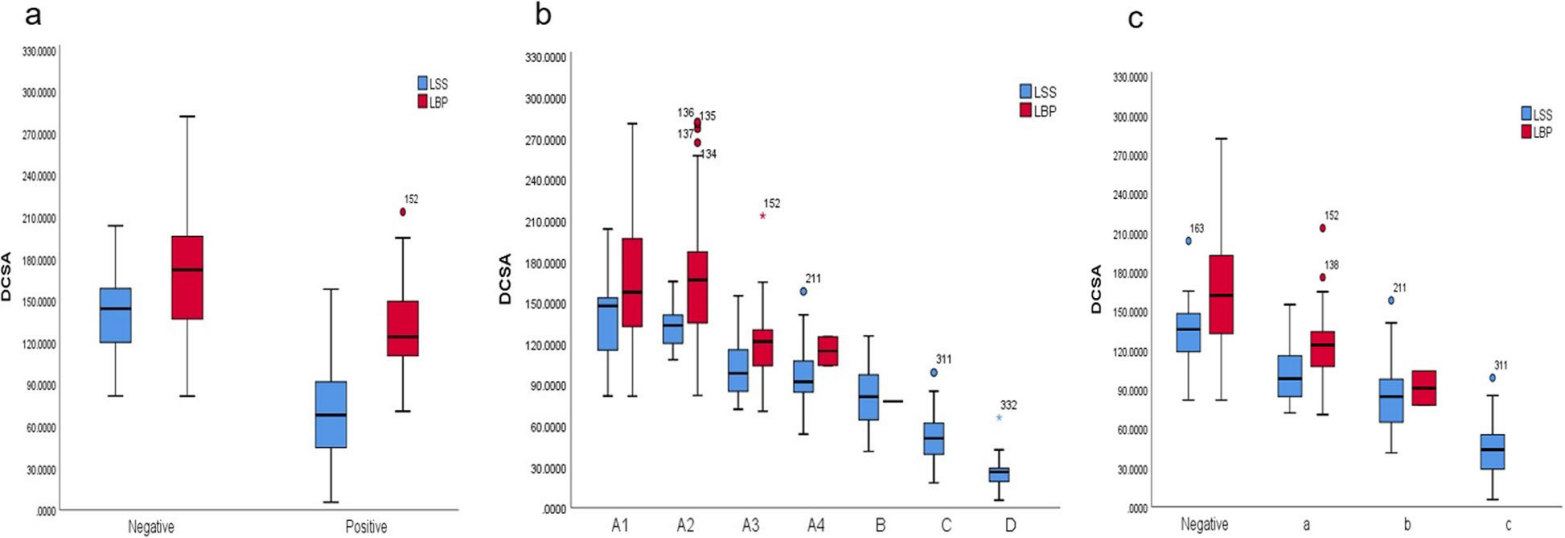
Boxplot of the three classifications for differentiating LSS from LBP at different levels of spinal stenosis. (**a**) Braz classification (**b**) Schizas classification (**c**) Chen Jia classification. The top and bottom lines of the box represent the 25th to 75th percentile values, the line in the box represents the median value, the lines outside the boxes represent maximum and minimum values and circles represent possible outlier values

For the diagnosis of LSS and LBP patients with different degrees of spinal stenosis, Braz classification showed no significant difference in the evaluation of mild spinal stenosis between LSS and LBP patients (*p* > 0.05). However, in the degree of canal stenosis it is found that there were significant statistical differences between Schizas and Chen Jia classifications (Fig. 4). Both Schizas and Chen Jia scores are significantly correlated with the degree of DCSA stenosis (*p* < 0.001). Chen Jia classification has the highest correlation with DCSA, which can more accurately evaluate the degree of spinal canal stenosis.

### The advantage of using clinical and imaging analysis for surgical indications

Table 5 summarizes the results of univariate and multivariate analyses of clinical and imaging data. Univariate models with only clinical variables showed that VAS (odds ratio [OR], 1.692; 95% CI: 1.106,2.589; *p* < 0.05), sex (OR, 0.843; *p* < 0.05), lower extremity pain (OR, 3.676; 95%CI: 1.291, 10.461; *p* < 0.05), and intermittent claudication (OR, 9.022; 95%CI: 3.562, 22.852; *p* < 0.001) are associated with surgical indication. The univariate model incorporating Chen Jia classification and DCSA into the imaging variables showed that Chen Jia classification (OR, 3.614; 95%CI: 2.321, 5.628; *p* < 0.001), DCSA (OR, 1.843; 95%CI: 1.318, 2.577; *p* < 0.001) are correlated with surgical indications. The combined clinical and imaging analysis showed that Chen Jia classification, DCSA and intermittent claudication are related to surgical indications.

**Table 5:** Clinical and imaging with LSS surgical indications univariate and multivariable analysis

	Clinical model		Imaging model		Combined clinical and Imaging model	
	*OR*	*p*	*OR*	*p*	*OR*	*p*
Chen Jia classification			3.614 (2.321,5.628)	< 0.001	2.127 (1.596,2.835)	< 0.001
DCSA			1.843 (1.318,2.577)	< 0.001	0.369 (0.169,0.802)	0.012
VAS	1.692 (1.106,2.589)	0.015				
Gender	0.457 (0.248,0.843)	0.012				
Lower limb pain	3.676 (1.291,10.461)	0.015				
Intermittent rupture	9.022 (3.562,22.852)	< 0.001			9.481 (3.439,26.142)	< 0.001

Numbers in parentheses are 95%; *OR*  odds ratio, *DCSA*  dural sac cross-sectional area, *VAS* visual analogu scale

## Discussion

In this study, among the three classifications, it is found that Chen Jia classification not only has high diagnostic efficiency in differentiating LSS and non-specific LBP, but also has the highest consistency with the degree of spinal canal stenosis. From the multivariate analysis, it is found that in addition to intermittent claudication, Chen Jia classification and DCSA can be used as a surgical treatment indication for patients with LSS, and the correlation between Chen Jia classification and the association of patients requiring surgery is higher than DCSA.

For many years, clinical symptoms and various imaging examinations have been used to diagnose LSS. It is difficult to base simply on clinical symptoms as the diagnostic standard for LSS. Hence the use of MRI to improve diagnosis has become the standard imaging method modality [15]. In the past, quantitative measurement on the transverse MR images, namely DCSA, is used in most cases for assessing the degree of stenosis [16]. However, this method has the disadvantages in that it is difficult to use in the clinical practice and that there are controversial results between the degree of spinal stenosis and clinical symptoms [17]. Therefore, NRSS received extensive attention when it was first proposed. Banitalebi et al. [18] reported that MRI findings of LSS is found to have a high degree of intra- and inter-observer consistency and in this study the intra- and inter-observer Kappa values of the three classification are all greater than 0.75, an indication that NRSS has high reliability and repeatability. A large number of studies also confirmed that NRSS has good diagnostic value for LSS [7–9, 19], but Zhang et al. [14] and Piechota M et al. [12] questioned the diagnostic value of Braz classification of NRSS for mild to moderate degree of LSS. In this study, the different Braz classification for mild spinal stenosis between LSS and LBP groups is not statistically significant, while Chen Jia classification and Schizas classification have good diagnostic efficacy in determining different degrees of spinal stenosis.

Macedo et al. [20] reported that the overall sensitivity of the NRSS Braz classification is 54%, and when LSS patients with DCSA < 80 mm^2^ and who has limited mobility are introduced to the study, the NRSS sensitivity increased to 82%. Since the patients included in this study have a definite diagnosis, the number of patients with LSS and non-specific LBP is comparable. Among the 176 patients with LSS, 99 patients have DCSA less than 80 mm^2^, only 19 patients have DCSA greater than 120 mm^2^. Among the156 patients with non-specific LBP, 132 patients have DCSA greater than 120 mm^2^, only 24 patients have DCSA less than 120 mm^2^, and only 3 patients have DCSA less than 80 mm^2^. Hence, we believed that NRSS can distinguish between LSS and non-specific LBP. It is noted that NRSS positive alone cannot diagnose LSS, but NRSS positive is an additional evaluation tool to support the diagnosis of LSS because of its good sensitivity and specificity. Among the three classifications of LSS, the diagnostic efficiency of Braz classification is relatively low, and Schizas classification has 7 different Grading, which are complicated and not very practical. Chen Jia classification has the best correlation with DCSA and is related to surgical indications, so it will be a more favorable classification method.

Zhang et al*.* [21] followed up the treatment effect of 62 patients with LSS according to Schizas classification, and concluded that conservative treatment for grade B patients can effectively shorten the average length of stay, reduce the economic burden of patients and save medical resources. Patients with grade C may undergo surgical treatment to alleviate the problems of poor effect and recurrent symptoms in conservative treatment. Studies [22, 23] have aslo shown that patients with positive nerve root sedimentation sign are more likely to benefit from decompression surgery, so morphological classification of the nerve roots of LSS patients on MRI-based transverse images can be used in guiding clinical treatment, which is beneficial to patient as they can receive timely and accurate treatment.

We note that there are some limitations in our current study. First, NRSS is limited to diagnosing central lumbar spinal stenosis and not lateral recess stenosis. Second, as L1-2 intervertebral disc is rarely imaged during MRI procedure, very few lesions are located in the L1-2 intervertebral disc and that in the L5-S1 intervertebral disc nerve roots do not deposit dorsally to the dural sac only L2-L5 intervertebral disc MRI were used in this work. Lastly, as this study is a retrospective study, there are difficulties in following up patients with postoperative spinal canal MRI and their treatment efficacy. In addition, there might be selection biasing in the inclusion of LSS and LBP patients to this study.

## Conclusion

To improve the accuracy in determining LSS and non-specific LBP patients, a combination of clinical symptoms and imaging examinations are required. All three classifications have good diagnostic performance, but in comparing their diagnostic accuracy and clinical practicability, Chen Jia classification will be the more favorable classification method. In addition, radiologists and clinicians may find that Chen Jia classification will be the better option in assisting them in classifying the severity of LSS and selecting the optimum treatment method for patient, which is beneficial for the patient as they will be able to receive more timely and effective treatment as well.

## Data Availability

All data generated or analysed during this study are included in this published article.

## Abbreviations

LSS: Lumbar spinal stenosis
DSCA: Dural sac cross-sectional area
NRSS: Nerve root sedimentation sign
MRI: Magnetic resonance imaging
LBP: Low back pain
CSF: Cerebrospinal fluid
ROC: Receiver operating characteristic curve
AUC: Area under curve
CI: Confidence interval
OR: Odds ratio
